# Deep-Learning-Assisted Focused Ion Beam Nanofabrication

**DOI:** 10.1021/acs.nanolett.1c04604

**Published:** 2022-03-24

**Authors:** Oleksandr Buchnev, James A. Grant-Jacob, Robert W. Eason, Nikolay I. Zheludev, Ben Mills, Kevin F. MacDonald

**Affiliations:** ‡ Optoelectronics Research Centre, University of Southampton, Highfield, Southampton SO17 1BJ, United Kingdom; § Centre for Disruptive Photonic Technologies & The Photonics Institute, SPMS, Nanyang Technological University, Singapore 637371, Singapore

**Keywords:** nanofabrication, deep learning, focused ion beam milling

## Abstract

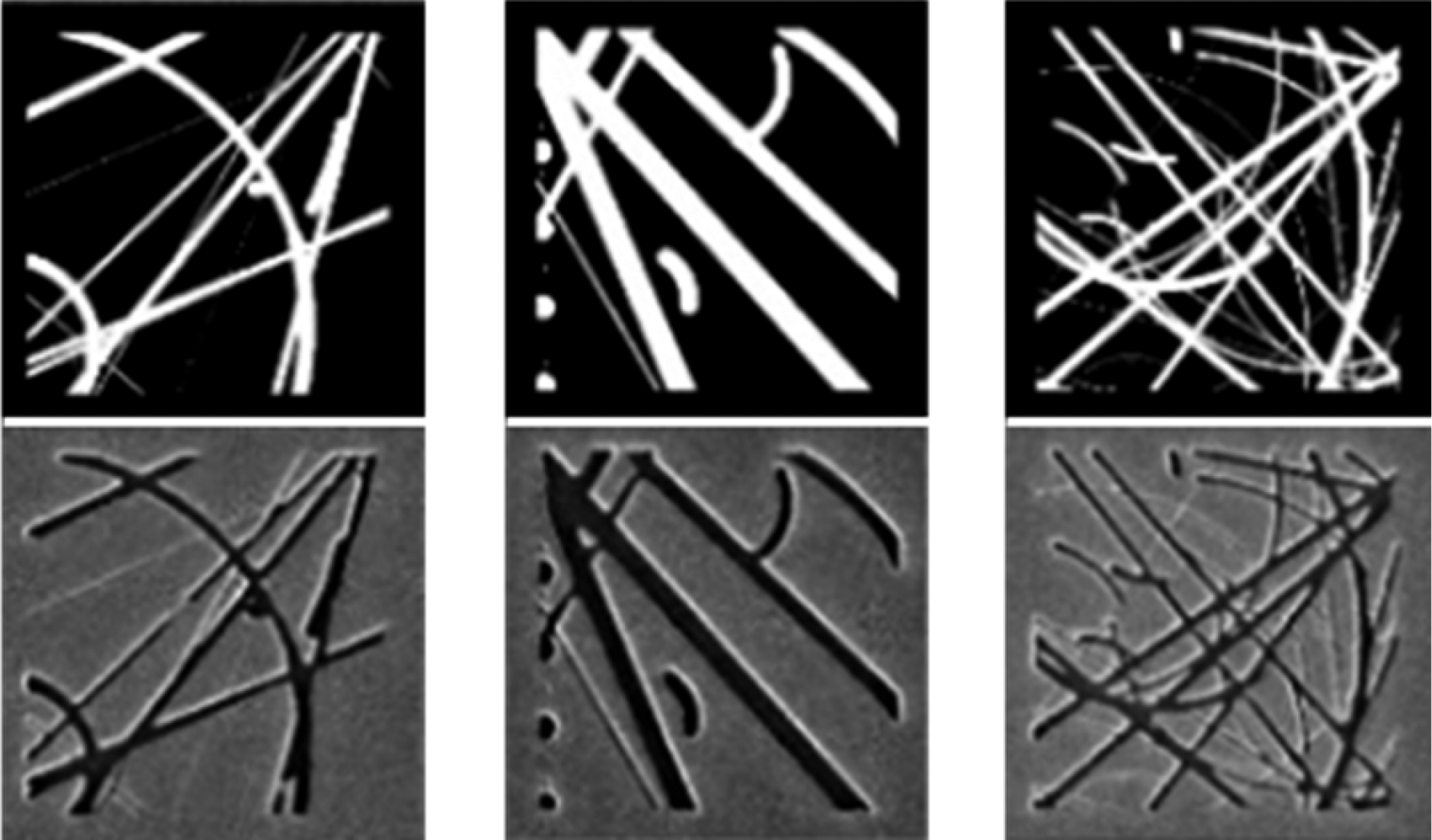

Focused ion beam (FIB) milling is an important rapid prototyping tool for micro- and nanofabrication and device and materials characterization. It allows for the manufacturing of arbitrary structures in a wide variety of materials, but establishing the process parameters for a given task is a multidimensional optimization challenge, usually addressed through time-consuming, iterative trial-and-error. Here, we show that deep learning from prior experience of manufacturing can predict the postfabrication appearance of structures manufactured by focused ion beam (FIB) milling with >96% accuracy over a range of ion beam parameters, taking account of instrument- and target-specific artifacts. With predictions taking only a few milliseconds, the methodology may be deployed in near real time to expedite optimization and improve reproducibility in FIB processing.

## Introduction

Focused ion beam (FIB) milling is a “direct-write” fabrication technique based on the removal of material from a target surface by a focused beam of ions.^1,2^ It can etch features with nanometric resolution into almost any metal, semiconductor, dielectric, or biomaterial. As such, it has become a standard tool in semiconductor (i.e., microelectronics) manufacturing, rapid prototyping, and nano/bio/materials research. The end result of any FIB milling process is a complex function of beam current, spot size, scan pattern, target material characteristics, and design geometry, especially the aspect ratio of the pattern. Producing a comprehensive analytical model that describes the physical processes occurring during milling is consequently an intractable problem. Time-consuming, trial-and-error testing is therefore invariably required to establish optimal process parameters for achieving the intended outcome of a given milling operation on a given target.

Computational, numerical approaches to simulating FIB milling are typically based on Monte Carlo modeling of ion–atom interactions and string or level set methods to track surface propagation over time.^3−8^ They can reproduce 2D and 3D cross-sectional profiles of FIB-milled trenches or holes in certain materials but are mathematically complex and again require detailed knowledge of numerous parameters, such as ion energy and angle of incidence to a target surface, ion flux, ion beam spot size and intensity profile, dwell time and raster step size, atomic mass and physical form (e.g., mono/polycrystalline; amorphous) of target, etc. Deep learning offers an alternative approach whereby milling outcomes for arbitrary nano/microstructural geometries in any target medium can be accurately predicted by a suitably trained neural network.

Deep learning is revolutionizing scientific research^9−14^ because of its aptitude for pattern recognition and the capability to empirically establish the functional algorithms of complex systems.^15^ For example, it has been shown that deep learning can improve laser machining processes,^16−18^ including through the provision of feedback for real-time process control.^19−21^ Here we show that deep learning can be used to simulate the postfabrication appearance of structures manufactured by FIB milling in the 2D projection of a scanning electron microscope image, as a very good (almost invariably the first, in situ) indicator of process accuracy and quality. With predictions generated on millisecond time scales, the approach can be deployed to reproducibility and precision in FIB manufacturing processes.

## Results and Discussion

**Figure 1:**
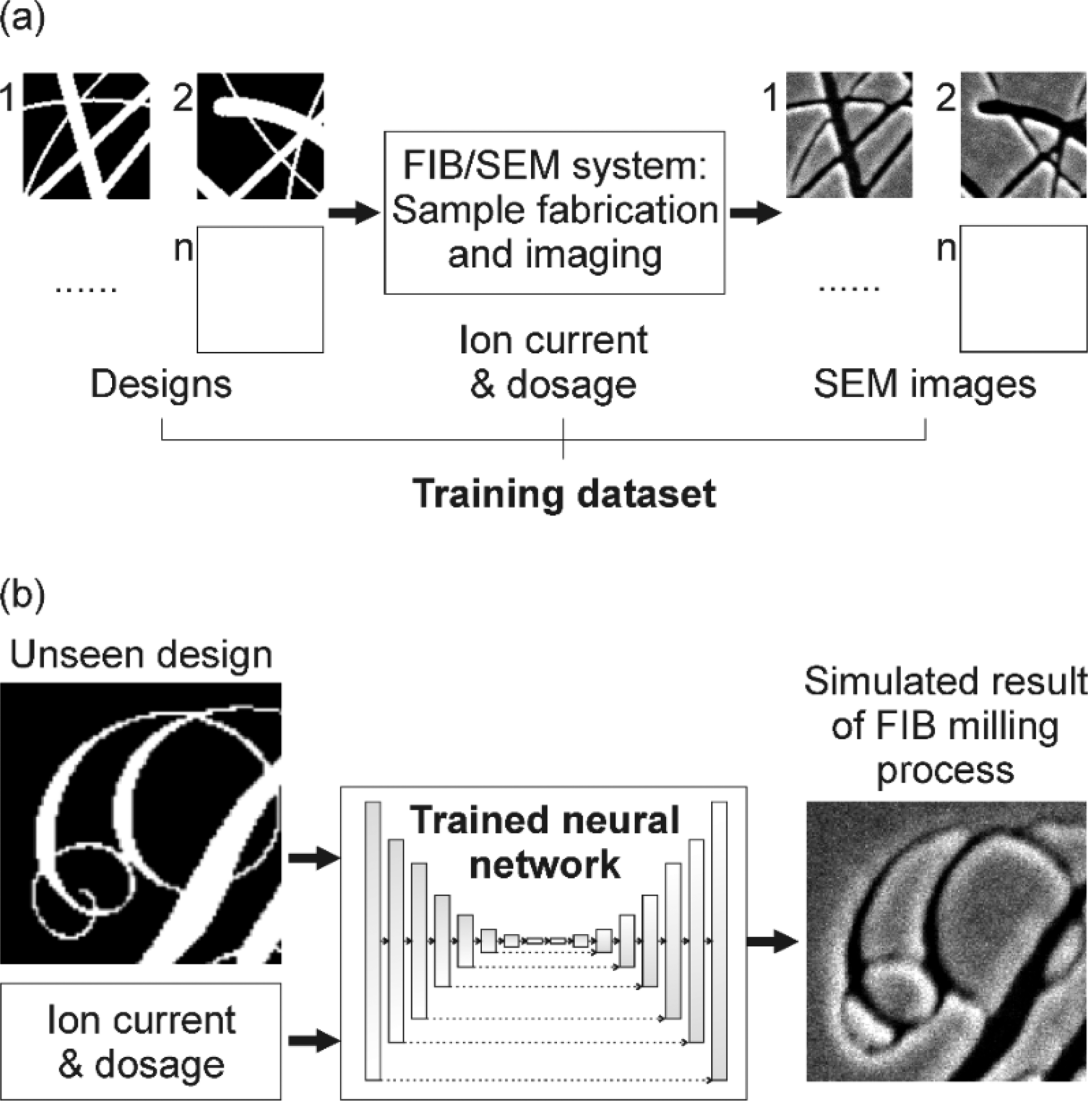
Deep learning simulation of FIB milling. (a) Neural network is trained on a set of binary design patterns, corresponding SEM images of samples manufactured by FIB milling, and detail of the ion beam parameters used in their production. (b) Trained network is then able to accurately predict the outcome of FIB milling processes—the expected postfabrication appearance of samples in SEM imaging—for previously unseen designs. Optoelectronics Research Centre “Light” logo used with permission.

We demonstrate that deep learning can assist in predicting the postfabrication appearance of two-dimensional binary patterns FIB milled into a gold thin film (Figure 1). Such structures are widely used in nanophotonic and metamaterial devices, seeded growth of nanostructures, and a range of other applications.^22−26^

In this work, we used an FEI Helios Nanolab 600 DualBeam FIB/SEM system, which incorporates a gallium ion gun with a milling resolution of ∼20 nm and a field-emission scanning electron microscope (SEM) with imaging resolution down to ∼1 nm. In all cases, the binary patterns (i.e., comprising areas either exposed or not exposed to the ion beam) were fabricated by raster scanning the ion beam in lines running from left to right (as seen in images below), stepped from top to bottom. This consistency of procedure, as will be demonstrated, is essential to the effective application of deep learning to prediction of process outcomes.

**Figure 2:**
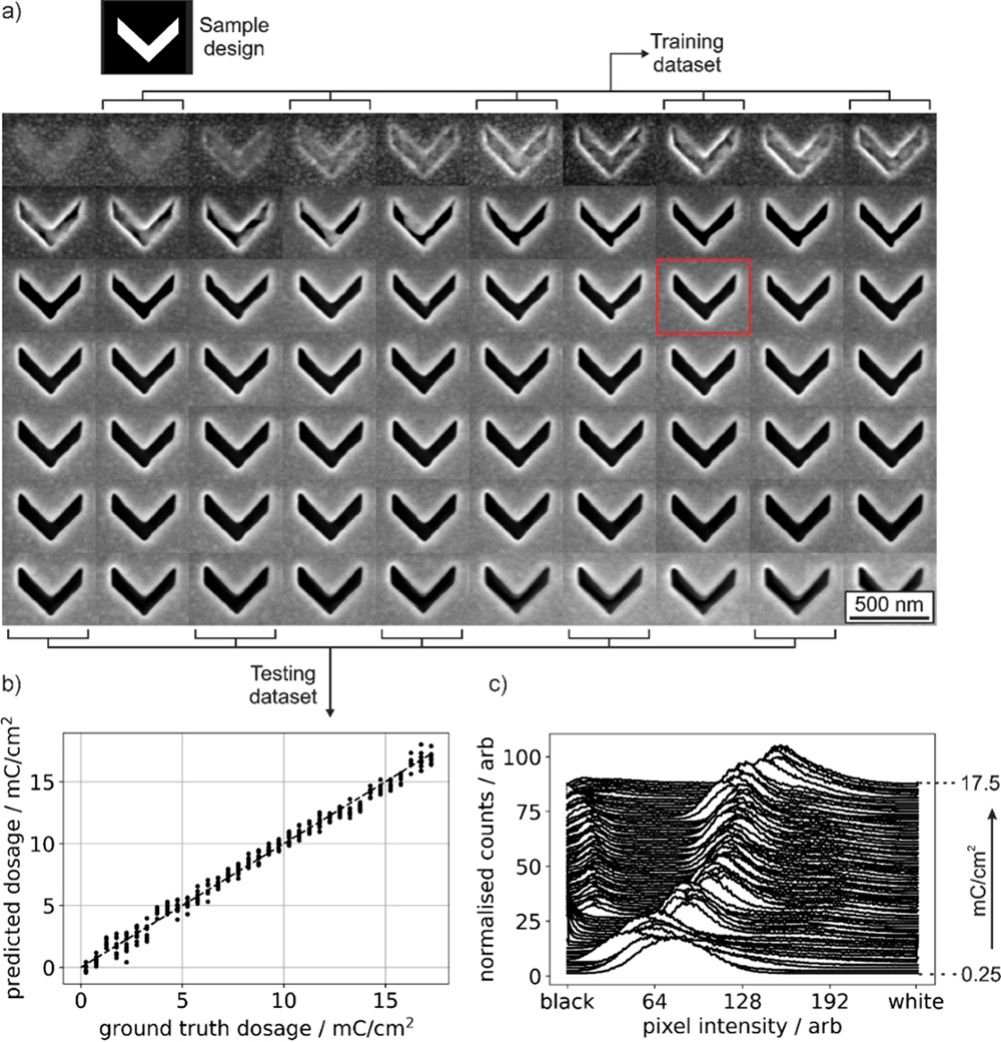
(a) SEM images of one of nine sets of 70 chevron structures milled into a 50 nm thick gold film with ion beam dosages ranging from 0.25 [top left] to 17.5 mC/cm^2^ (bottom right). The chevron design (i.e., input pattern for FIB milling) is shown above. Half of the images (as labeled) are used for neural network training; the other half for testing (panel b). Later experiments (see Figures 3–5) use a dose of 7 mC/cm^2^, as per the chevron image outlined in red. (b) Correlation between trained neural network predictions of mC/cm^2^ milling dosage from chevron SEM images in the testing set and actual (ground truth) dosage used in fabrication of the sample. (c) Histograms of pixel intensity for the set of chevron SEM images shown in panel a (peak count number amplitudes are normalized for clarity).

We first demonstrate the use of deep learning to relate the appearance of a simple geometric design—an isolated submicron chevron shape (Figure 2a)—in an SEM image to the ion beam parameters employed in its fabrication. To create a training and testing data set, we milled the chevron shape into a 50 nm thick (thermally evaporated) gold film nine times at 70 different FIB dosage settings, ranging from 0.25 to 17.5 mC/cm^2^ in 0.25 mC/cm^2^ steps, with a fixed ion beam current of 9.8 pA, creating 630 separate chevron elements. Figure 2a illustrates how the chevron’s appearance in SEM imaging changes with increasing ion beam dosage, from the top left (where it is insufficient for the design to penetrate the full thickness of the gold film) to the bottom right.

A convolutional neural network (CNN, see the Supporting Information) was trained using one half of the experimental SEM image data set and tested on the other. Figure 2b shows experimental versus predicted dose for the 315 images in the testing set; there is a clear correlation, with a root mean squared error of 0.52 mC/cm^2^. Although the method of dosage identification established by the neural network cannot be known, the set of pixel intensity histograms in Figure 2c provides some insight into the statistical differences among SEM images of chevrons milled with different ion beam settings: There is a clear trend toward higher pixel intensities (i.e., brighter images) with increasing dose, and a small peak at low pixel intensity emerges when the dose is high enough for the design to be milled through the full thickness of the gold film, exposing the silicon substrate (which appears black in SEM images).

We next addressed the more complex challenge of simulating the FIB milling process for arbitrary patterns, aiming to accurately predict the outcome, i.e., what a sample will look like in an SEM image (as a strong indicator of process accuracy and quality), for previously unseen designs.

**Figure 3:**
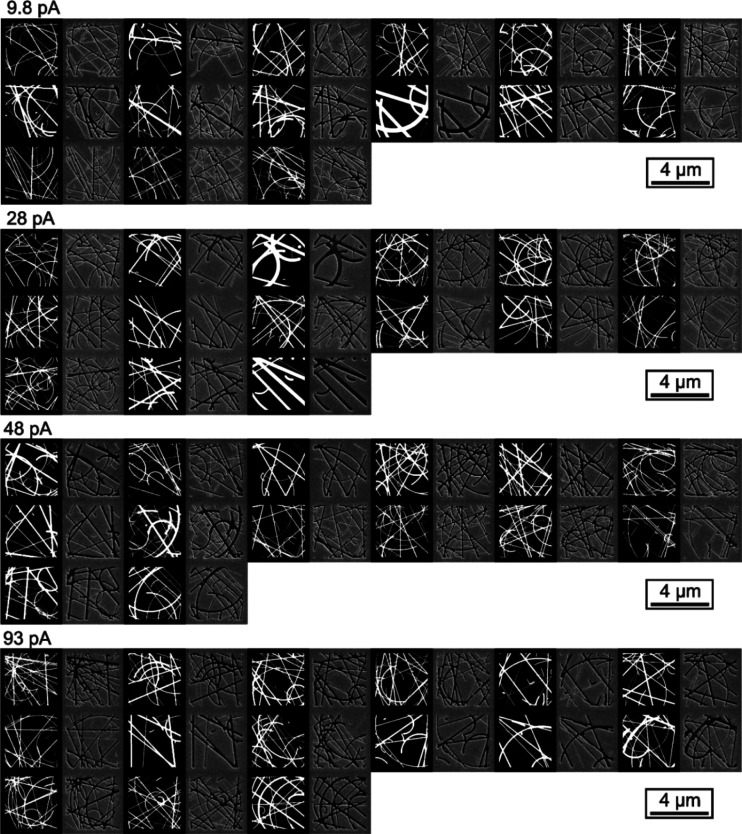
Training data set for FIB process simulation by a neural network, showing randomly generated binary designs (left of each pair) and corresponding FIB-milled sample SEM images (right), grouped by the ion beam current setting employed in sample fabrication.

This simulation is performed by a conditional generative adversarial network (cGAN)^27−29^ trained on a set of 59 binary design and corresponding sample SEM images (Figure 3, see the Supporting Information for detail of the adversarial configuration used in network training). Designs comprised randomly generated arrangements of straight line and circle segments (each of random length and line width), so as to collectively include a very wide variety of straight/curved edges and intersection angles, at assorted orientations to the ion beam raster scan direction. They were fabricated using one of four ion gun aperture (beam current) settings with a fixed dosage of 7 mC/cm^2^ (sufficient to consistently penetrate the gold film without excessive overmilling of the substrate, as illustrated for the chevrons in Figure 2a by the highlighted SEM image).

**Figure 4:**
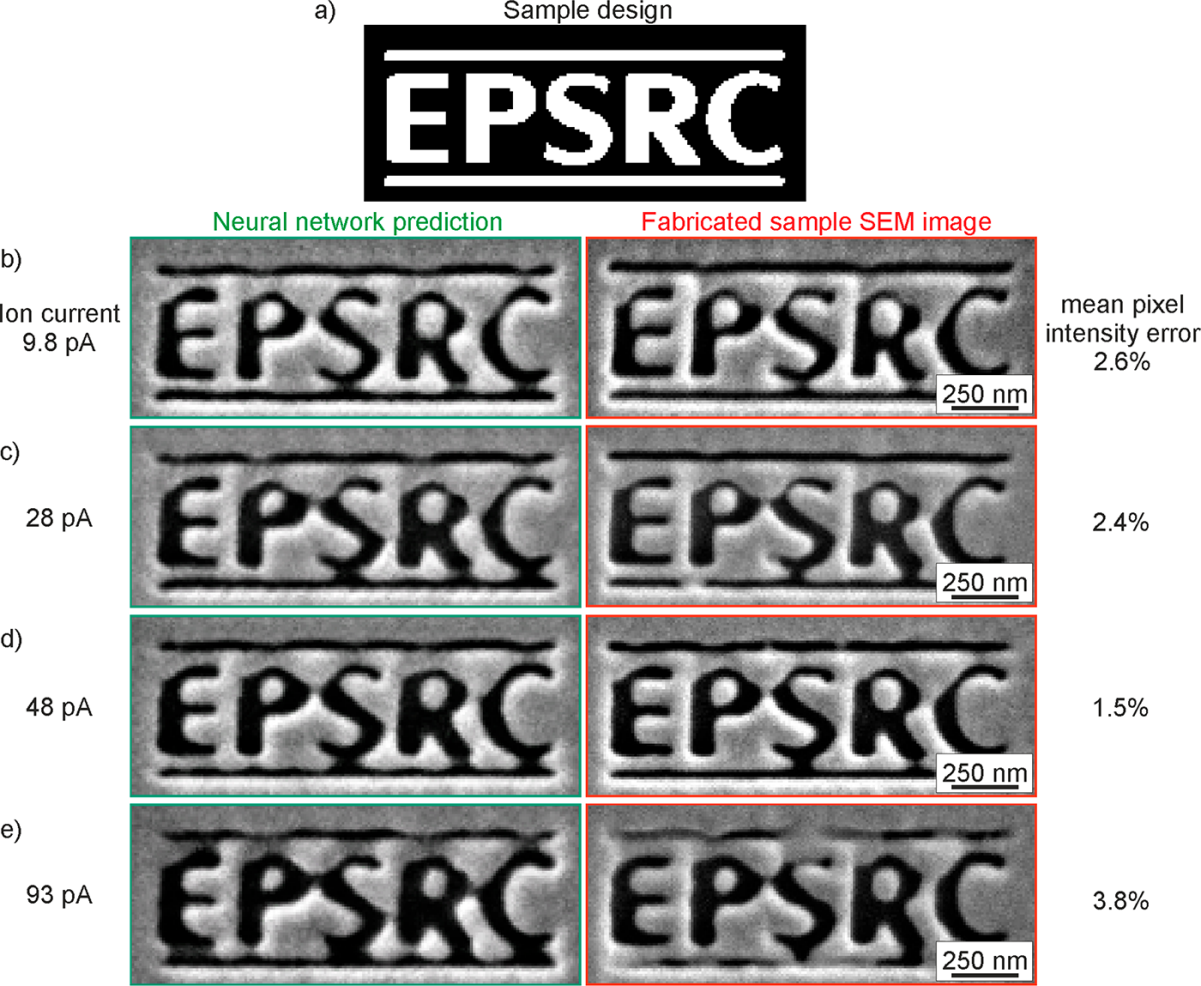
Comparison between neural network-predicted (left column) and actual FIB-milled sample SEM images (right column) for the EPSRC logo: (a) the binary design, and images with ion beam current (aperture) settings of (b) 9.8, (c) 28, (d) 48, and (e) 93 pA. Engineering and Physical Sciences Research Council (EPSRC) logo used with permission.

The trained network was subsequently asked to predict what SEM images of previously unseen binary designs would look like, were they to be fabricated with certain ion beam settings. Figure 4 shows a comparison between predicted and actual FIB milling process outcomes for the acronymic logo of the UK’s Engineering and Physical Sciences Research Council at submicrometer font size (character height ∼360 nm). The accuracy of neural network predictions is evaluated here in terms of the mean magnitude of pixel intensity difference between predicted and real sample SEM images, above the noise level intrinsic to SEM imaging (i.e., of an unstructured surface). The correlation is extremely good, with the network achieving >96% accuracy over the entire trained order-of-magnitude ion beam current range: with increasing beam current, for example, increased overmilling of the design is correctly predicted, such as in the gradual disappearance of the gap between the letters P and S. Remarkably, the network correctly predicts defective shaping in the top left corner of each letter that is consistently observed across all ion current settings. This is an artifact (likely related to ion beam alignment and/or raster scan pattern) that has been identified by the network as systematically present within the training data set, while being imperceptible there to the human eye. The network has then accounted for this process artifact in its predictions of milling outcomes.

**Figure 5:**
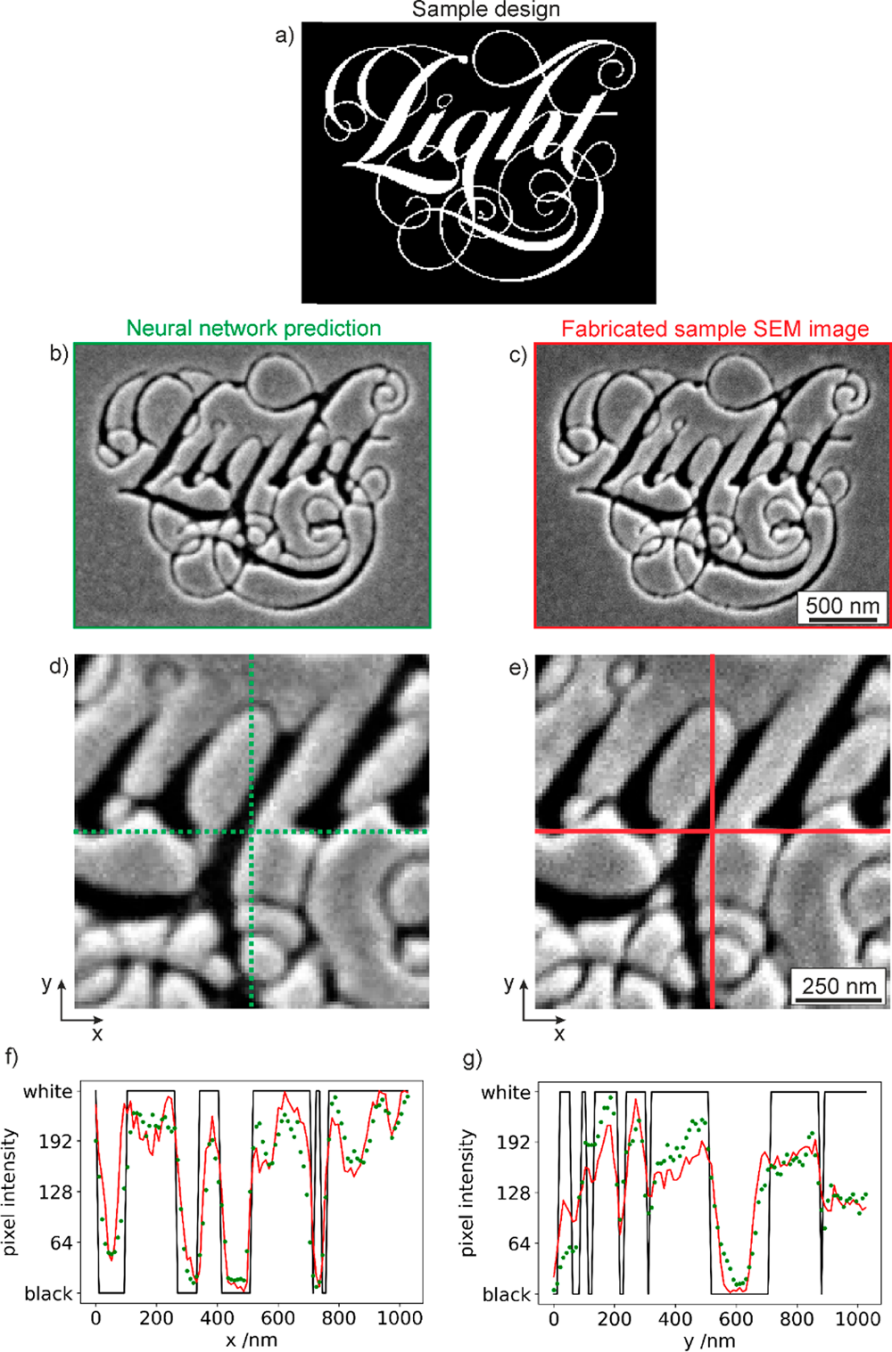
(a) Binary design image of the Optoelectronics Research Centre’s “Light” logo. (b) Neural network-predicted (left column) and (c) actual FIB-milled sample SEM images of the logo, for an ion beam current setting of 9.8 pA. (d, e) Enlarged detail of central 100 × 100 pixel regions of b and c. (f, g) Pixel intensity profiles along the (f) horizontal and (g) vertical green and red lines in panels d and e, plotted in those colors (i.e., network-predicted profiles in green; fabricated sample SEM image profiles in red). The overlaid black lines are the corresponding binary design profiles (inverted with respect to panel a as white areas of the design are milled, becoming black in experimental samples). Optoelectronics Research Centre “Light” logo used with permission.

Figure 5 presents a more detailed, quantitative comparison between predicted and experimental images of our departmental “Light” logo, which features an assortment of curved and intersecting lines of varying width, akin to the training patterns. The prediction accuracy for the image as a whole (i.e., evaluated as above, as the mean magnitude of pixel intensity difference between predicted and real sample SEM images, above the unstructured surface noise floor) is >98%. Cross-sectional pixel intensity profiles through corresponding parts of the design provide insight into the network’s success in meeting its objective: It is tasked, in essence, with mapping the binary (input) profiles denoted by black lines in Figure 5f, g to the experimental sample profiles from SEM imaging (plotted in red), with a success metric being the difference between the latter and the dotted green lines, which show network-generated profiles. Even though the transformation is both highly nonlinear and dependent on the surrounding pixel intensities in all directions, there is strong correspondence over a range of feature sizes in the design between predicted and experimental sample profiles. Improvements in this correlation may be achieved through optimization of the training hyperparameters (e.g., number of epochs and learning rate; see further detail in the Supporting Information) and/or inclusion of additional training data, for example, to address circumstances in which the network’s predictive capability is found to be weak.

## Conclusion

In summary, we have shown that a neural network can accurately predict the postfabrication appearance in scanning electron microscope images of samples manufactured by focused ion beam milling, over a wide range of sample design geometries (arbitrary micro/nanostructural feature shapes and dimensions), and ion beam parameters (current and per-unit-area dosage). With each prediction taking only a few tens of milliseconds, this capability can significantly reduce the time and the number of experimental dose-test iterations required in the development and optimization of new FIB processes It can also be employed to rapidly evaluate the impact of design or process parameter modifications, and to maintain performance (i.e., consistency of outcomes from established processes) against aging of the ion source and ion gun beam apertures, thereby increasing the useful lifetime of said components, particularly where employed in highly repetitive (e.g., cross-sectional characterization) tasks. Predictions are sufficiently accurate as to include instrument- and/or target material-specific artifacts. These cannot be accounted for in numerical or analytical approaches to simulating FIB milling. That they may be imperceptible to the human eye in training samples, or may appear as random (e.g., redeposition) defects in isolated test samples, raises the prospect that such networks could be deployed for early fault (e.g., beam alignment, aperture damage) detection and identification.

In this proof-of-principle study, we trained a network to simulate a specific type of FIB milling task on a specific target medium, while varying ion current and dosage (i.e., keeping all other system parameters constant). In practice, one would train the network to the task(s) at hand (i.e., according to application context, such as in semiconductor wafer-based device characterization or in nanofabrication for plasmonics research), on a relevant variety of target materials, and with a full range of substrate and system metadata (e.g., film deposition methods, rates and thickness, crystal orientations, etc.; ion current, dosage, raster scan pattern, number of repetitions, ion source, aperture age, etc.). In this way, the network would accrue an “understanding” of the complex relationships among the numerous sample and system parameters that affect process outcomes.

Indeed, we would argue that there is considerable scope for functional enhancement of FIB/SEM systems, as integrated micro/nanomanufacturing and sample characterization (i.e., fabrication and in situ diagnostic) platforms, through the application of machine learning methodologies. For example, we (as chemists, physicists, and materials scientists and as users of FIB milling tools) understand that families of materials have similar physical properties derived from similarities in composition and atomic/molecular structure. Neural networks are highly effective at discovering such patterns in complex, multidimensional data sets can similarly “learn” that there are relationships among types of material.^30−34^ Thus, a network trained on, for example, conductive oxide compositions A, B, C, and D may “intuitively” perform very well in application to previously unseen composition E, if told simply that it is another conductive oxide, i.e., it would not require dedicated training on every new target material encountered. It is also possible that neural networks trained for applications to FIB process development and control could contribute to new scientific understanding of the milling process (i.e., ion beam–target interactions).^15,35−37^

## Supporting Information Available

Further detail on convolutional neural network (CNN) and conditional generative adversarial network (cGAN) architectures (PDF)

## Notes

Following a period of embargo, the data from this paper will be available from the University of Southampton ePrints research repository: 10.5258/soton/D2030.
