# Supplementary material for: Deep-Learning-Assisted Focused Ion Beam Nanofabrication
Source: Nano Lett. 2022 Mar 24;22(7):2734–9. doi: 10.1021/acs.nanolett.1c04604 (PMC9097578; doi:10.1021/acs.nanolett.1c04604)
Supplement: Supplementary file 1 — nl1c04604_si_001.pdf [file nl1c04604_si_001.pdf]

Deep Learning-Assisted Focused Ion Beam Nanofabrication

Oleksandr Buchnev[*], James A. Grant-Jacob[*], Robert W. Eason[*], Nikolay I. Zheludev[*] [†], Ben Mills[*], and Kevin F. MacDonald[*]

** Optoelectronics Research Centre, University of Southampton, Highfield, Southampton, SO17 1BJ, UK*

† Centre for Disruptive Photonic Technologies & The Photonics Institute, SPMS, Nanyang Technological University Singapore, 637371, Singapore

Convolutional Neural Network (CNN) for predicting ion beam dose from sample SEM images

The CNN was constructed of four convolutional layers, each formed of batch normalization, ReLU (Rectified Linear Unit) and pooling processes[1-5], followed by a fully connected layer with a single regression output using a mean square error loss[5].

The network was trained on SEM images of chevrons milled with dosage values in the set [0.5, 1.0, 1.5, …. 17.5] mC/cm^2, cropped to 299×299 pixels with random vertical and horizontal shifts of ±15 pixels in the position of the chevron relative to the center of the frame. Training proceeded for 50 epochs, with a learning rate of 0.00005 and batch size of 8, taking 3 minutes on an NVIDIA RTX2070 GPU.

In more detail, the input image was 299×299×3, followed by convolutions of 8 of 3×3×3 (with stride of 1), 16 of 3×3×8 (with stride of 2), 32 of 3×3×16 (with stride of 1), and 32 of 3×3×32 (with stride of 1).

The CNN was then tested on SEM images of chevrons milled with dosage values in the set [0.25, 0.75, 1.25, … 17.25] mC/cm^2, cropped to 299×299-pixels, with each prediction taking 2.5 ms.

Conditional Generative Adversarial Network (cGAN) for simulation of FIB-milled sample SEM images

The cGAN was formed by an 8-layer generator and a 4-layer discriminator, with a L1 to discriminator loss ratio of 200, learning rate of 0.0002, and was trained for 5 epochs on an NVIDIA Titan Xp GPU (taking 45 hours).

The network was trained on a set of fifty-nine 355×355-pixel binary design (input) and corresponding FIB milled sample SEM (output) images, and detail of the ion gun aperture (beam current) setting used in sample fabrication (Figure 3).

The SEM images were recorded at 710×710 pixels with a resolution of 5 nm. These were then rescaled by a factor of two, to 355×355 pixels (with 10 nm pixel size), matching the pixilation of the binary designs.

The dataset was augmented through random cropping of the 355×355-pixel images down to 256×256 pixels.

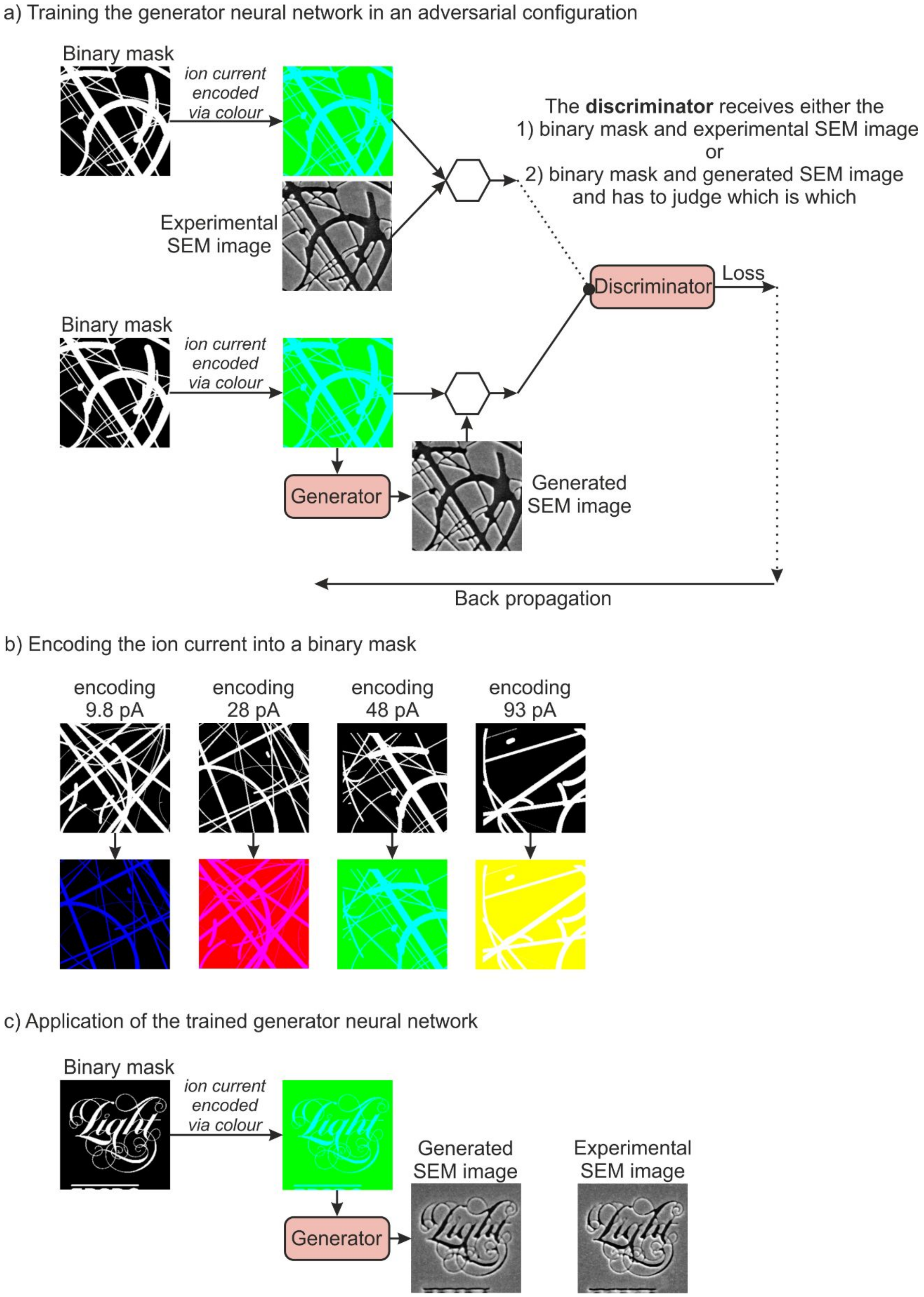

Figure S1. Schematic of the cGAN for adversarial training of the generator neural network used for predictive visualization. Optoelectronics Research Centre "Light" logo used with permission.

After training, the cGAN was able to predict the appearance of a sample for any desired binary map, with each predictive visualization being computed in only 85 ms.

Figure S1 illustrates the training and application of the cGAN in more detail. Figure S1a shows a schematic of the generator and discriminator configuration, where the generator network transforms a binary mask into a generated SEM image, and the discriminator judges whether the generated SEM image appears experimental or generated. The expectation of this adversarial training approach is that, under appropriate conditions, the generator and discriminator networks become more effective over time.

In this work, the four different ion currents were included in the training data by changing the colour theme of the initial black-and-white binary masks, as shown in Fig. S1b.

As shown in Fig. S1c, once training was complete, the generator network could be used to transform any binary mask into a generated SEM image, i.e. as shown here, and in the manuscript, for transforming previously unseen binary mask images, which were not used during the training process.

References

1. Le, Q. V.; Jaitly, N.; Hinton, G. E. **2015**, arXiv:1504.00941v2.
2. Nair, V.; Hinton, G. E. In *Rectified Linear Units Improve Restricted Boltzmann Machines*, 27 th International Conference on Machine Learning, Haifa, Israel, 2010; Haifa, Israel.
3. Canziani, A.; Paszke, A.; Culurciello, E. **2016**, arXiv:1605.07678v4.
4. Chen, G.; Chen, P.; Shi, Y.; Hsieh, C.-Y.; Liao, B.; Zhang, S. **2019**, arXiv:1905.05928.
5. Miao, S.; Wang, Z. J.; Liao, R. *IEEE Transactions on Medical Imaging* **2016,** 35, (5), 1352-1363.
